# What establishes an excellent nurse? A focus group and Delphi panel approach

**DOI:** 10.1186/s12912-017-0239-x

**Published:** 2017-08-10

**Authors:** Wolter Paans, Patricia Robbe, Inge Wijkamp, Marca V. C. Wolfensberger

**Affiliations:** 1 Research Group Nursing Diagnostics, Hanze University of Applied Sciences, Groningen, the Netherlands; 2 Research center for talent development in higher education and society, Hanze University of Applied Sciences, Groningen, the Netherlands; 3 School of Health care Studies, Hanze University of Applied Sciences, Groningen, the Netherlands

**Keywords:** Delphi panels, Education, Excellence, Focus groups, Nursing care

## Abstract

**Background:**

Over the past few years, the complexity of the health care system in which nurses are required to practice has increased considerably, magnifying the need for excellent professionals with a specific set of knowledge, skills and attitudes. However, the characteristics that distinguish an excellent nurse have not yet been clarified.

The aim of this study was to determine nurses’ perspectives regarding characteristics associated with an excellent nurse in order to elicit a conceptual profile.

**Method:**

A focus group design followed by Delphi panel content validation was utilized. Information regarding nurses’ perspectives was derived from six focus group discussions comprising 19 nurses involved in hospital practice and 24 nurses with experience in mental health care. The analysis of the focus group discussions resulted in nine domains whereby content validity was achieved with contributions from a Delphi panel survey with 26 professionals.

**Results:**

As determined by the survey, a combination of these specified aspects characterize an excellent nurse: analytical, communicative, cooperative, coordinating, disseminates knowledge, empathic, evidence-driven, innovative and introspective.

**Conclusion:**

Determining what establishes an excellent nurse according to experienced nurses is valuable as this information can influence the broadening curriculum for educating future nurses to meet the needs in the professional field, contributing to the quality of care. This conceptual profile can be used as a reference guide for supervisors and professionals to personally improve their clinical practice as well as for education.

## Background

Over the last few years, the health care domain has changed considerably. Nurses face an aging population that is experiencing co-morbidity and multi-morbidity as well as intensifying care requirements. Not all nurses believe they are equipped for these challenges [1]. To deal with complex health care situations, an increased level of knowledge and the development of expert skills appear to be required [2]. Also, the changing health care domain requires more innovative behavior of professionals, and requires nurses who are able to generate new ideas, and implement them to achieve outstanding accomplishments [3]. To answer these needs in the professional field, the interest in identifying excellent professionals and training talented students to achieve professional excellence has become an increasingly important topic for both hospital administrations and universities [4].

Accordingly, many nursing schools are launching so called honors programs. Honors programs are selective study programs that are designed for motivated and gifted students who want to accomplish more than the regular program offers [4, 5]. These programs have clear admission criteria and clear goals, and offer educational opportunities that are more challenging and demanding than regular programs [4, 5], aiming at positively influencing (nursing) students’ growth toward professional excellence [5, 6]. In the Netherlands, these honors programs are considered as the testing ground for innovations, in which motivated and talented students can experiment with new challenging educational methods, that are often implemented in the regular program, when feasible [7, 8].

The designation ‘excellent nurse’ has not been distinctly specified in literature nor has it been evidenced how the concept is interpreted in the field of nursing. As the construct lacks content validity, it can neither be applied for clinical practice nor for education. The term ‘excellence’ connotes explicit and, supposedly, superior attributes, behaviors, pursuits, and nursing status and is associated with knowledge, skills, and experience [9]. The lack of research towards the conceptualization of ‘excellence in nursing’, necessitates referring to opinions. One of the first authors indicating the importance for refining the concept ‘excellence in nursing’ was Virginia Henderson [10]. Henderson links excellence in nursing to ‘the potential for intellectual, emotional or spiritual growth’. Excellence in nursing, according to Henderson, is not seen as the generic definition ‘to excel, to surpass, or to be the best’. Coulon et al. (1996) [11] refer to professionalism, holistic care, good practice, and humanism. Enabling personal qualities, nurse-patient relationships and nurse-health team relationships can be seen as subcategories for identifying excellence in nursing.

Benner [12] uses the term ‘expert’ as an equivalent of ‘expertise’ in a development model for nurses whereby nurses are facilitated in their progression from ‘novice’ to ‘expert’. Benner describes the five levels of nursing experience as: 1. Novice; 2. Advanced beginner; 3. Competent; 4. Proficient; and 5. Expert. According to Benner, not all nurses exhibit the required competencies that are needed to become an expert [12]. Possessing an ability to intuitively employ tacit knowledge based on experience are key traits according to Benner’s theory. Sternberg [13, 14] and Dweck [15] state that communication, leadership, and decision-making skills as well as teamwork, situational cognizance, metacognitive knowledge, social recognition, self-esteem, confidence, and flexibility are significant disciplines for developing expertise. Diers and Evans [6] mention aspects such as being reflective, acting with an internal drive, and having a focus on other disciplines as being associated with outstanding or excellent performance. A cross-sectional survey study among nurses (*n* = 159) in China revealed that greater social boldness, receptivity to change, self-reliance, dominance, and vigilance can be construed as predictors of what makes an excellent nurse [16]. Furthermore, core principles such as dignity, respect, compassion and person-centered care are key issues associated with providing excellence in nursing care [17]. Sternberg [13, 14] states that expertise comprises two aspects, i.e., “a cognitive and an attributive aspect”. The cognitive aspect is related to the automation of complex tasks. Attribution is what is perceived or experienced as expertise. Therefore, only demonstrating professional knowledge and skills is not sufficient enough to provide excellent nursing care [12, 18–20]. A study among allied health care professionals has established that demonstrating outstanding competencies in only one particular specialty area does not mean the equivalency of being excellent. An excellent caregiver can be an expert, however a broader, in depth, inter-professional orientation on an outstanding level must also be present [20]. Ericsson [21–23] and Sternberg [13] describe expertise as possessing a ‘superior competence level’. ‘Excellence’ is also identified as a concept related to ‘professional art and science’ in clinical practice [24–28]. The concept of professionalism also seems to be related to professional excellence. According to Tanaka [29], professionalism refers to the conduct, qualities, or goals that characterize a profession and usually describes behaviors that are highly expected of the profession’s members.

Based on definitions and descriptions in literature, it remains ambiguous if being an ‘expert’, having ‘expertise’, or exhibiting an ‘outstanding competence level’ or ‘professionalism’ can be designated as antecedents or are synonymous to what is construed as an ‘excellence nurse’. In addition, to our knowledge, the analysis of how nurses perceive the concept ‘excellent professional’ is lacking in the current literature. Therefore, we conducted a study to investigate which characteristics distinguish an excellent nurse, according to experienced nurses, in order to establish a framework to guide talented nursing students in their development towards professional excellence and satisfying the expectations of professionals in the clinical nursing environments.

## Methods

### Purpose

The focus of the study was to identify the characteristics of an excellent nurse from the perspective of experienced nurses in order to compile a conceptual profile. This profile can be beneficial as a reference tool for nurses in clinical practice and can function as a reference in the development of learning outcomes for educational programs aiming to guide students toward becoming an excellent nurse. Thereby, a profile can lend support to teachers in nursing schools for developing honors education curricula and for students’ self-reflection.

### Focus group design

A focus group design according to Hennink, Hutter & Bailey [30] was employed to investigate experienced nurses’ perspectives and opinions on the competencies that distinguish an excellent nurse. The elucidation of perspectives during group discussions may expose information that would not have surfaced during in-depth interviews conducted in individual meetings or in a survey [30]. A topic guide was structured in order to discern detectible characteristics (performance, knowledge, attitude, disposition, interference) exhibited by an excellent nurse. The main question proposed in the focus groups was: ‘What do you believe establishes an excellent nurse in your health care environment?’ Additional questions included: ‘Is there a difference in what is considered to be an experienced, well-educated, or specialized nurse and an excellent nurse?’; ‘What is your opinion regarding the differences between an expert nurse and an excellent nurse?’; ‘What is the difference between an excellent nurse and a regular nurse, in your opinion?’

### Sample and recruitment

Focus group participants resided in the Netherlands and were selected by remitting emails to members of a university-nursing network within a hospital and a mental health care facility and by snowball sampling. The focus group settings included: 1) three focus groups with clinical hospital nurses and 2) three focus groups with nurses employed in mental health care. The group composition in segmented groups of hospital and mental health care nurses was meant to create homogeneity in the degree of shared experience of the discussion topic as similarity in background fosters an open and productive discussion among participants [30]. To be able to generate enriched data, we included nurses from various wards and health care institutions with a variety of background experiences and specialties within the segmented groups. This resulted in dynamic sessions and afforded the collection of a diversity of perceptions and opinions. Participants were included in accordance with the following inclusion criteria: (1) employed in a clinical hospital practice, at a mental health care institution, or at a health center for psychotherapy as a registered nurse and (2) consent to participate in a focus group discussion regarding personal opinions and perspectives on what constitutes an excellent nurse for a maximum duration of 90 min. Nurses were provided with information beforehand regarding the essence of the research project as well as the methods utilized. Contact information of the research team was included in the event the participants had inquiries regarding the content of the study.

Six focus group (FG) discussions were arranged in February–March 2013 with a convenience sample of 19 hospital nurses (FG 1: *n* = 7, FG 2: *n* = 5, FG 3: *n* = 7) and 24 nurses with experience in mental health care (FG 4: *n* = 9, FG 5: *n* = 8, FG 6: *n* = 7) comprising a total of 43 participants in the study (Table 1). In FG 1–3, nurses from surgery wards, internal medicine wards, neurology, critical care, and a pediatric ward were included. In FG 4–6, nurses in psychiatric emergency units as well as those in extended-stay psychiatric settings were included. Nurses involved in mental health care were experienced caregivers in extramural psychiatric settings as well as in intramural environments with diverse patient groups (i.e. schizophrenia, bipolar mood disorder, borderline, depression, anxiety disorders, and forensic psychiatry). The mean (standard deviation) age was 37 (12) *n* = 33 (from a total of 43 nurses, 33 nurses indicated their age). Seventy-five percent of the focus group participants were female. All participants had acquired more than five years of experience in their field.

**Table 1:** Nurses in focus groups

FG 1–3: Hospital Nurses (*n* = 19)	
FG 1	*n* = 7
FG 2	*n* = 5
FG 3	*n* = 7
FG 4–6: Mental Health Care (*n* = 24)	
FG 4	*n* = 9
FG 5	*n* = 8
FG 6	*n* = 7

### Data collection

Each of the six focus groups discussed the topic “characteristics that differentiate an excellent nurse” led by a moderator and monitored by a non-participating observant. The principal investigator was one of the two observers who participated in three out of the six focus groups. The moderator exploited the topic guide to foster the conversations and encourage the participants to proportionally contribute to the deliberation. All focus group discussions were audio recorded. The observers transcribed the field notes aggregated during the interactions within the focus group. Two discussions occurred in a hospital unit, two in an institution for mental health care, and two on a university campus.

### Analysis of focus group discussions

Audio recordings were transcribed verbatim. Two researchers (the principal investigator and one observer) independently performed data analyses. Each precisely examined the text and repeatedly correlated text analyses to create a preliminary profile with the characteristics distinguishing an excellent nurse as derived from the participants’ perceptions. The continuous cycle of aggregating and analyzing data followed the grounded theory approach described by Hennink, Hutter & Bailey [30]. This method was employed in order to provide analytic rigor in the interpretation of the qualitative data and when developing empirical theory.

Data analysis was initiated with open coding which involved disseminating the data into distinct units of analysis and classifying the dissimilar units as concepts. Concepts were labeled, whenever feasible, by utilizing the terms conveyed by the participants (i.e., in vivo coding). Axial coding was subsequently performed in order to attain a greater degree of data conceptualization. To attain this goal, both researchers independently created items and domains which were then compared until consensus was reached. The analysis of textual data was realized by utilizing the qualitative analysis software package ATLAS.ti, version 06 (2012) [31].

### Delphi survey for content validity

To validate the findings of the focus group sessions and arrive at a quantifiable concurrence of the characteristics that comprise an excellent nurse, a survey tool was created based on the results of the focus group discussions. The survey was in accordance with the Delphi technique as indicated by Lawshe [32] and consisted of domains and items that distinguish an excellent nurse to be rated on a three-point scale. Experts (*n* = 52) that were employed in health-related professions received an invitation to participate in the Delphi survey. Participants included senior nurses with a specialization in the development of expert skills, senior researchers, honors educators and publishers in the field of nursing care and nursing care education. Professionals participating in the focus groups were excluded. This slightly expanded inclusion criterion was used in the Delphi panels compared to the focus groups as we expected that senior researchers as well as teachers involved in honors education could be considered as experts in the field of excellence in nursing, even though they were not necessarily primarily involved in nursing practice.

Content-validity ratios were quantified to assess the level of consensus in each Delphi panel. Each panelist was queried as to whether or not the domains and aspects indicated in the preliminary profile that discerned an excellent nurse were actually essential for this profile. Domains alluding to an agglomeration of attitudes, characteristics, or the aspects that collectively constituted a category, and aspects in the preliminary profile, i.e., related professional behaviors, were also measured in accordance with the method of Lawshe [32] and by indicating the following scale anchors: “essential”; “important, but not essential”; or “not necessary”.

The following formula specifies this measurement:

$$ CVR=\frac{ne-\mathrm{N}/2}{\mathrm{N}/2}, $$

in which CVR is the content-validity ratio, ne symbolizes the number of panelists who assessed a specific item or domain as “essential”, and N is the entire number of panelists.

It was requested that the panelists communicate their opinions in two phases concerning the material of the concept profile. Phase I pertained to the results of the focus group; Phase II was applicable to the modifications derived from the CVR calculation of Phase I. Only items and domains that acquired positive ratios were included in the conceptual profile.

A similar method was utilized to ascertain the distribution of the components within the domains. Panelists were requested to evaluate the accuracy of the positioning of the items on a four-point Likert scale where −1- signifies a correctly located item and −4- indicates that an item must be situated within a different domain. Panelists judged an item as being incorrectly categorized resulting in relocation more than 50% of the time.

Phase I of the Delphi panel comprised 27 respondents, and Phase II consisted of 26 respondents from the 52 experts who were contacted (response rate, 52% and 50%, respectively). All of the respondents satisfied the inclusion criteria whereby they were regarded as experts in the realm of nursing based on whether they were an author of publications in the field of policy; a researcher in nursing, nursing care, or nursing education; and/or possessed substantial leadership positions in nursing. All participants had at least bachelor-level education and at least five years of professional experience in nursing practice. Of the participants in Phase II, 77% (20) were female. Nineteen of them indicated their age of which the mean (standard deviation) was 42 (6) years.

### Ethical approval and consent to participate

In the Netherlands, ethical concession is not required for studies exploiting a focus group comprising nurses and an anonymized data analysis as this is only required in patient related research and not in that considered to be professional. The written invitations to the professionals requesting them to participate in the study also incorporated statements conveying confidentiality and privacy procedures, the prerogative to revoke participation at any time, and a declaration that the sessions would be audio recorded. All of the participants provided informed oral consent and requested that any personal information discussed within the focus groups remain confidential. All data that may plausibly have identified participants were eliminated from the transcripts to ensure anonymity.

## Results

### Results of focus group discussions

An iterative process as described by Hennink et al. [30] identified the point of information saturation. Variations in the issues decreased following the assessment of the third focus group session, and aggregation of the data continued in order to determine whether any further information emerged. Data saturation was also achieved pursuant to the assessment of the third focus group session in both groups, i.e., mental health care nurses and hospital nurses, as no additional issues appeared during this session compared to previous sessions in both health care sectors.

The text analysis generated domains describing a theoretical schema based on the experiences, perceptions, and interpretations that were communicated by the participants. The codebooks of the focus group sessions from the hospital and the mental health care nurses were compared representing the analysis of the transcripts. As no considerable variations were verified between the codebooks of discussions with hospital nurses and mental health care nurses, one single conceptual profile of the “characteristics of an excellent nurse” was generated.

### Results of the Delphi panels

The Delphi panel results supported the generated conceptual distinctions, and only negligible remarks were made regarding the focus groups results. ‘Experienced’ was considered “not necessary” in the profile of what comprises an excellent nurse. Although the concepts ‘experience’, ‘proficiency’, ‘occurrence’, ‘accurate skills’, ‘well-understanding’ and ‘knowhow’ were referenced in the discussions several times, there was no consensus in the focus groups or in the Delphi panels that these concepts are perceived as characteristics that distinguish excellence but, instead, are considered normal nursing traits. Other terms appointed in the focus groups as well as in the Delphi panels as ‘being above average curious’, ‘showing more perseverance’, ‘energetic’, ‘enthusiastic’, ‘spirited’, or ‘above average thinker’ were also not considered essential for the profile as they were regarded as not concrete, tangible, and distinguishing perceptible characteristics in terms of performance, knowledge, attitude, disposition, interference.

A minority (<50%) of the panelists regarded “networking” (ratio [r] = 00 (13/26) and “publishing in professional or scientific journals” (*r* = − 46 (7/26) as indispensable elements in the profile of an excellent nurse. Therefore, it was determined that these items should be omitted from the profile. In response to the qualitative analysis of the comments from the Delphi panelists, several items were rephrased to be less ambiguous and more functional in nature.

**Fig. 1:**
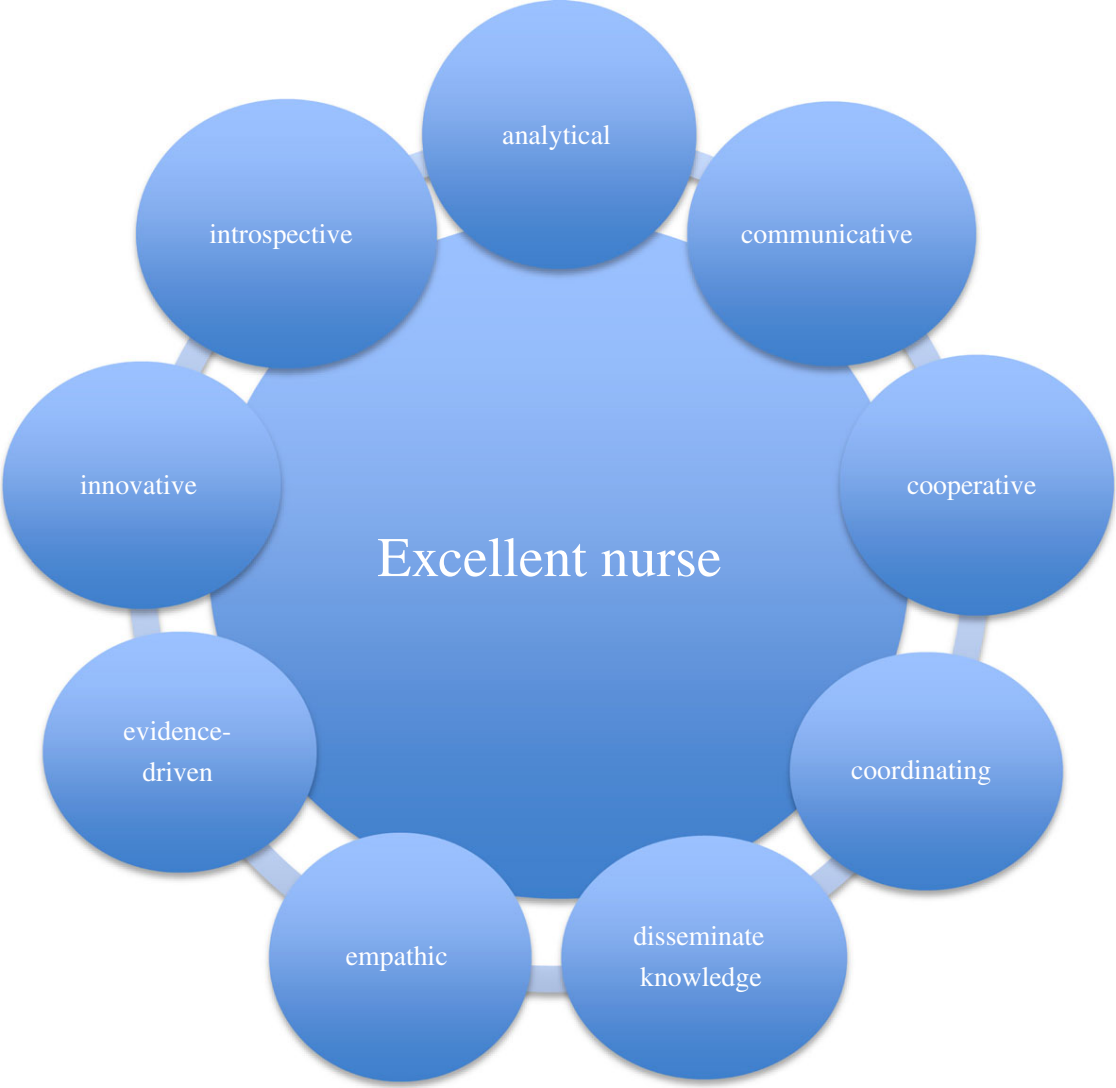
Conceptual profile of what establishes an excellent nurse

Following the second Delphi Phase, all items were placed in the appropriate domain as indicated by more than 50% of the panelists who assessed the location of the items in the domains as correct (Fig. 1).

### The profile of the excellent nurse

By compiling the conclusions from the textual analysis of the focus group discussions followed by content validity through the calculation of both Delphi panel judgment phases (Table 2) and the qualitative interpretation of the Delphi panel observations (Fig. 1), the conceptual profile of an excellent nurse was established. This conceptual profile comprises nine attributes or characteristics, listed in alphabetical order: (1) analytical, (2) communicative, (3) cooperative, (4) coordinating, (5) disseminate knowledge, (6) empathic, (7) evidence driven, (8) innovative, and (9) introspective.

**Table 2:** Domains and characteristics of what establishes an excellent nurse

Domains and characteristics of what establishes an excellent nurse	Ratio (n/N) (judged as “essential” by 26 experts in Delphi round two)
Analytical	0.69 (22/26)
Effectively Analyzing complex patient situations	0.85 (24/26)
Able to effectively distinguish main issues from side issues in complex situations	0.85 (24/26)
Exhibiting a comprehensive perspective in complex situations (a bird’s eye view)	0.85 (24/26)
Diagnosing patient needs and effectively initiating action	0.77 (23/26)
Making sensible decisions quickly in complex situations	0.69 (22/26)
Communicative	0.77 (23/26)
Communicating clearly and relevantly	0.77 (23/26)
Communicating the results of one’s professional interactions	0.77 (23/26)
Using context-sensible expressions	0.46 (19/26)
Cooperative	0.85 (24/26)
Taking the lead in complex situations	0.77 (23/26)
Inspiring and encouraging others to work effectively together	0.62 (21/26)
Taking the lead in general teamwork	0.62 (21/26)
Focusing on cooperating with professionals in various settings	0.54 (20/26)
Coordinate	0.69 (22/26)
Advocating: taking personal responsibility for the organizational choices in favor of the patient	0.77 (23/26)
Accurately transferring patient sensitive information	0.69 (22/26)
Leading other professionals to the right place at the right moment	0.38 (18/26)
Disseminate knowledge	0.92 (25/26)
Sharing knowledge in an interdisciplinairy context	0.92 (215/26)
Sharing knowledge in an multidisciplinary context	0.69 (22/26)
Using knowledge in a broad organisational context to improve quality of care in patient groups	0.69 (22/26)
Sharing knowledge with the patient	0.62 (21/26)
Developing new professional knowledge	0.38 (18/26)
Empathic	0.38 (18/26)
Connecting to the individual patient situation and patients’ experiences	0.69 (22/26)
Treating the patient with full respect in every situation	0.62 (21/26)
Evidence-driven	0.92 (25/26)
Applying principles of evidence based practice	1.00 (26/26)
Advancing opportunities to change or to improve interventions based on scientific evidence	0.92 (25/26)
Seeking and discovering scientific knowledge	0.69 (22/26)
Researching what are the best nursing interventions in clinical practice	0.39 (16/23)
Innovative	0.54 (20/26)
Initiating new and more efficient approaches in work processes	0.77 (23/26)
Proactively implementing nursing care innovations in practice	0.62 (21/26)
Encouraging others to generate new ideas and alternatives	0.54 (20/26)
Advancing opportunities for improvement	0.46 (19/26)
Developing creative alternatives in complex situations	0.23 (16/26)
Introspective	0.69 (22/26)
Using feedback to stimulate personal accomplishment	0.92 (25/26)
Self-reflecting on one’s own professional approach	0.77 (23/26)
Reflecting to be able to make the most appropriate situational fit and to use context-sensible expressions	0.77 (23/26)
Trying continuously to improve, seeking for self-development	0.69 (22/26)
Keeping within the limits of one’s capabilities	0.62 (21/26)

#### Analytical

Employing critical thinking skills to analyze complex patient situations in order to ascertain a precise overview of the situation was mentioned in all of the focus groups. Possessing the ability to distinguish primary issues that are based on critical reasoning skills in complex situations from those that are incidental was recognized in all focus groups as well. One participant indicated: ‘In complex situations, seeing the big picture, and, by this time, doing the right thing, where I am still asking myself, ‘What to do now?’ [FG2].

#### Communicative

The issue of ‘being communicative’ was referred to in all six focus groups. In regard to the transcript analyses, the term ‘Communicative’ was coded with the highest scores. It was discussed being a normal nursing skill as well, though it was perceived as a characteristic of an excellent nurse and related to employing context-sensible expressions [FG1–3, FG6]. ‘Relevant communication’ [FG3,4] was mentioned several times. Communication was often associated collectively with ‘providing clear argumentation for interventions’ [FG1,2,5,6]; ‘correctly explaining the things you do’ [FG3–6]; and ‘vibrant communication to share knowledge’ [FG3,4]. ‘An excellent nurse is always a good communicator; without high quality communication, one cannot be excellent’ [FG1].

#### Cooperative

Being cooperative was regarded as being a normal nursing skill. Cooperation in combination with ‘taking the lead in complex situations’ was discussed as a characteristic of excellence in four out of the six focus groups [FG1–3,6]. Collaboration in the context of an excellent nurse was considered as being an ‘outstanding and inspiring team worker’ [FG2]. ‘Supporting colleagues when they need it in no matter what subjects’ [FG2].

#### Coordinating

Being cooperative and an accurate coordinator was often used in the same context of ‘taking the lead’. Whereas the term ‘cooperation’ was considered from a team perspective, the term ‘coordinating’ was seen from the patient objective although ‘accurately transferring patient sensitive information’ refers to cooperation as well as coordination. Coordination is perceived as ‘managing patients’ preferences in an efficient clinical pathway’ [FG1].

#### Disseminate knowledge

Sharing knowledge with patients, nurses, and other significant professionals to improve the quality of care was referred to in all focus groups. ‘Having knowledge or experience is not what makes a nurse excellent; enlarging your professional knowledge continuously, and sharing knowledge does’ [FG5].

#### Empathic

Being empathic was regarded as being essential by the six focus groups. ‘Every nurse needs to be empathic’ [FG1–6], and the capability of being professional and empathic in the most stressful situations can be considered as being oriented towards excellence. Being empathic and possessing a holistic approach often appeared in the same context. ‘Having polite and empathic expressions while doing the right things under high pressure; that’s excellent’ [FG3].

#### Evidence-driven

Improving opportunities to change or ameliorating interventions based on scientific evidence is considered as a trait of ‘higher educated or excellent nurses’ [FG3] and not to those who are more conventional. Exploiting scientific papers is generally not perceived as a normal nursing responsibility. ‘Locating the facilities to research nursing interventions that are determined as the best in clinical practice was, at times, associated with nurses’ career opportunities for the higher educated, outstanding ones’ was mentioned in one of the focus groups, further explaining: ‘In our institution, career chances in nursing science are achievable only by the excellent ones’ [FG5].

#### Innovative

Initiating new and additional more efficient approaches in the work process and not primarily complying with protocols or all day work schedules can be regarded as being associated with excellence in nursing and was proposed in all of the focus groups. Innovation is rarely mentioned in the context of inventing and implementing novel materials, however, working with new materials in innovative procedures as an invigorating pioneer was indeed related to excellence.

#### Introspective

Self-reflecting on capabilities, searching for opportunities of personal improvement, and using feedback to stimulate accomplishment can be an indication of excellence according to all focus groups, i.e., ‘being able to reflect objectively on harsh critique’ [FG2] and ‘not to see it (criticism) personally but professionally’ [FG4]. Reflectivity was determined to be a significant personal characteristic for every nurse but is most often recognized in those that are excellent.

### Excellent nurse versus expert

Participants in all of the focus group discussions agreed that a nurse can be an excellent professional without necessarily being an expert. The concept of being an expert is related to, being a specialist and to years of experience, according to the participants. A remarkable quote in this context is: ‘an expert can be an expert for a long time in one or two specialties, an excellent nurse always goes for an environment with challenges, new situations, and will never be bored’ [FG6]. Participants concurred that nurses are not required to have extensive experience to ask themselves: ‘Am I doing the right thing by using this protocol in this situation, or is this protocol outdated?’ However, a combination of experience and knowledge was considered as a precondition for nursing leadership. According to participants, excellent nurses strive to gain experience in a shorter period of time and learn quickly from others to afford performing in the same resourceful manner as experienced nurses. They do not strive for this in order to be competitive but, instead, to be prepared in the future and to take responsibility.

## Discussion

Based on the analysis, 36 items in nine domains were defined as characterizing an excellent nurse whereby a conceptual profile was created and referred to as “characteristics of an excellent nurse” (Fig. 1). The results of our study are generally in accordance with those of Higgs & Tichen [7] and Alsop [24] in that being reflective and analytical as well as sharing knowledge are considered characteristics of outstanding professionals. However, our delineations of what constitutes an excellent nurse are more detailed and specific. Moreover, several different opinions compared to literature were discovered.

An excellent nurse responds and performs appropriately in complex situations (e.g., stressful or unexpected situations) by employing an ensemble of particular competencies. Importantly, being empathic and having good communication skills were found to be essential for professional excellence in all six focus groups. In accordance with our findings, studies using empirical analyses of nurses and patients data also present effective communication and affective or interpersonal competence as key elements of quality nursing care [11, 33–35]. Based on their findings, Johnston & Smith [34] indicate the necessity of mandatory interpersonal skills training for nurses who work in palliative care. In light of our study, we could broaden this recommendation for nurses involved in different wards of clinical hospital practice and mental health care. Sepasi et al. [36] describes professional excellence as being related to the power of achieving goals and contributing to the development of the profession. Corroborating our findings, the authors also report that in the perception of nurses, being cooperative, inspiring colleagues, disseminating knowledge and aiming to improve quality of care contribute to the feeling of power and consequently professional excellence [36].

Comparing our results to Benners’ development theory of becoming an expert nurse, participants were not of the opinion that experience is one of the most important issues as a novice nurse can be excellent as well [12]. According to our study, even as a beginner with a minimal level of experience, characteristics can be combined from the previously described nine domains. Participants’ opinions revealed that ‘spending a long time in nursing’ is more a predictor for an expert nurse rather than for an excellent nurse. Furthermore, participants indicated that a nurse specialist (i.e., a master educated nurse practitioner) can be an expert in a specialty area but not a superior coordinator or communicator and would, therefore, not be considered as an excellent nurse. On the contrary, a novice who has just completed nursing school may be able to combine characteristics distinctive of excellence, such as acting reasonably and empathically in rather complex clinical situations, and exchanging knowledge ‘as second nature’ [37, 38].

In nursing, the field of excellence has been under study since the early 1970s. Several lists of competencies are related to nurses. However, our study delivers a concise list established in the view of nurses themselves. As we look, for instance, to the domains ‘communicative’, ‘cooperative’ and ‘introspective’, one can argue that competencies included in these domains can be seen as ‘regular skills’ and not explicitly referring to excellence. The ‘excellence factor’ might be found more clearly in an individual’s personal combinations, patterns and interrelationships of determinants among the domains and characteristics of the conceptual model, and not solely in the examination of each determinant. The conceptual profile might be beneficial when conducting research in larger groups that may provide more insight into strong combinations of competencies to be able to examine the concept ‘excellence in nursing’ more in depth.

Our focus group participants did not discuss in depth whether the features that characterize an excellent nurse could specify a nurse as ‘gifted’ or ‘outstanding’ and who is intelligent and capable by nature. According to Sternberg, a superior intelligence quotient (IQ) is not what makes one excellent. Cognitive and social expertise can be learned to some extent and is required in high quality job functioning [39]. The awareness of this assumption related to talent and excellence is of importance in higher education, especially in talent programs, such as honors programs, which are forthcoming in Europe and aiming to support student nurses to be able to use their talents and realize their full potential [40, 41].

The results of our study may be important for comprehending how to invest in nursing staff in order to deliver improved quality care. It may also afford an opportunity to identify those nurses with a potential for excellence at an earlier stage [37, 42]. For instance, the profile can be used as a reference for supervisors and professionals in the development of nurses in clinical practice. In educational settings, this profile may function as a guide against which students can evaluate themselves in the development of their professional identity. Future research is required to establish how the profile of an excellent nurse can be applied, for example, as a reflective model for students and novice professionals as well as a tool for curriculum development by guiding the development of learning goals in educational programs aiming for professional excellence.

### Limitations of the study

This study was limited due to using a convenience (snowball) sample and only focusing on two specialties in nursing (hospital nurses and nurses in mental health care) within one country. Therefore, it was not feasible to estimate possible international and/or cultural dissimilarities in the findings which may limit the transferability. Furthermore, our study reflects the characteristics that distinguish an excellent nurse according to experienced nurses. Given that contemporary health care is focuses on patient- and family-centred care, excellence in nursing should also be defined from the patient and family perspective. Future research including these stakeholders may reveal different or additional competences that distinguish excellent nursing care.

## Conclusion

Educating future nurses who are able to provide superior quality of care has become crucial due to the increasing complexity of the health care system in which nurses are required to practice. Experienced nurses and experts in the field of nursing regard competences related to the following characteristics as distinctive for an excellent nurse: analytical, communicative, cooperative, coordinating, disseminate knowledge, empathic, evidence-driven, innovative, and introspective. Establishing the characteristics distinctive for an excellent nurse positively influences the development of personal competences and professional identity, thereby increasing the quality of nursing care. It serves as a meaningful guideline for the development of educational programs in supporting talented students on their path of becoming the excellent nurse of the future. Educational practices based on guided reflection and a profile of what establishes an excellent nurse as an element of a nursing curriculum may inspire students to broadly improve their attitudes towards the goal of becoming an excellent nurse. This is of special interest for honors programs which aim to guide students to become the excellent professionals of the future. Moreover, this profile can play an important role in supporting talent development of regular students and professionals in health care institutions.
